# Looking for pyromania: Characteristics of a consecutive sample of Finnish male criminals with histories of recidivist fire-setting between 1973 and 1993

**DOI:** 10.1186/1471-244X-5-47

**Published:** 2005-12-14

**Authors:** Nina Lindberg, Matti M Holi, Pekka Tani, Matti Virkkunen

**Affiliations:** 1 Department of Psychiatry, University of Helsinki, Finland

## Abstract

**Background:**

As pyromania is a rare diagnosis with questionable validity, we aimed to describe a forensic psychiatric population of arson recidivists.

**Methods:**

The medical records as well as the forensic psychiatric examination statements of 90 arson recidivists referred for pretrial psychiatric assessment in Helsinki University Hospital Department of Forensic Psychiatry between 1973 and 1993 were reviewed.

**Results:**

The most important diagnostic categories of arson recidivists were personality disorders, psychosis and mental retardation, often with comorbid alcoholism. In all, 68% of arsonists were under alcohol intoxication during the index crime. Psychotic as well as mentally retarded persons with repeated fire-setting behaviour were mostly "pure arsonists"- persons guilty only of arsons during their criminal careers. Arson recidivists with personality disorder, in contrast, often exhibited various types of criminal behaviour and arson appeared to be only one expression of a wide range of criminal activity. Comorbid alcoholism was apparently a more rarely observed phenomenon among pure arsonists than in "nonpure arsonists". We found only three subjects fulfilling the present diagnostic criteria for pyromania.

**Conclusion:**

Using the criteria of the DSM-IV-TR, pyromania must be regarded as an extremely rare phenomenon. Especially the question of substance intoxication as an exclusion criterion for pyromania should be reconsidered.

## Background

Arson is a major source of property damage, injury and death in many Western countries. The incidence of arson appears to have increased both in the United States and in Europe in recent years [1,2] and in Finland it has doubled between 1985 and 1995 [3].

Fire-setting recidivism rates have varied widely in the literature, from 4% to as much as 60%, depending on the study population [4]. Mentally disordered fire-setters have higher rates of recurrence of fire-setting than nonmentally disordered fire-setters and commit fewer common offences other than fire-setting [5]. In the study by Barnett et al. [6] arsonists who were only partly responsible and who committed no crimes other than arson showed the highest number of fire-setting incidents. Arson recidivists have rarely been thoroughly characterized, but their psychiatric disorders appear to be heterogeneous and include schizophrenia, bipolar disorder, substance abuse, personality disorders as well as mental retardation [7-9]. Pyromania – deliberate and purposeful fire- setting on more than one occasion- is a rare phenomenon that seldom explains repeated fire-setting behaviour [10,11], and the phenomenon as a valid diagnosis has even been questioned [12]. Taken together, further research is needed to examine the different subgroups of arsonists [4].

We aimed to characterize arson recidivists by describing some major psychiatric and demographic variables as well as criminal histories of a representative sample of Finnish male criminals with repeated fire-setting behaviour. We further aimed to characterize arsonists with "pyromanic" behavioural patterns by extracting the "pure arsonists" i.e. those with only arsons in their criminal histories from the study population and comparing them with "nonpure arsonists" i.e. those with also other types of offences in their previous criminal careers. We hypothesized that these two populations may differ in that the mental disorders of pure arsonists would somehow explain their repeating behaviour whereas arsons committed by nonpure arsonists may have more instrumental properties.

## Methods

### Subjects

The hospital records and forensic psychiatric examination statements of arsonists who underwent a pretrial forensic psychiatric evaluation during a 21-year period (1973 – 1993) in Helsinki University Central hospital were reviewed. Ninety (22.4%) of the altogether 401 arsonists (all male over 16 years old) were recidivists i.e. had committed two or more separate arsons before the evaluation. Some of the participants were convicted in court for arson earlier during their criminal carrier, and others were in court process for the first time in their lives.

### Diagnostic categories

Psychiatric classification according to the International Classification of Diseases- Eight Revision (ICD-8) [13] was used in clinical practice in Finland between 1969 and 1986 and it was replaced by the classification according to ICD-9[14] in January 1987.

We pooled the principal diagnoses of 90 arson recidivists into 6 main diagnostic categories: mental retardation, psychosis, personality disorders, alcoholism, organic brain disorders and mood disorders. The diagnosis of mental retardation was based on an IQ of 70 or below [15,16]; 70% of the offenders in this category were proposed by the investigating forensic psychiatrist to be nonresponsible for the index crime while 30 % showed diminished responsibility. None of the mentally retarded persons guilty of repeated arsons were diagnosed as psychotic. Persons with the following ICD-8 diagnoses: schizophrenia paranoid type, schizophrenic residual state, schizophrenia catatonic type, schizophrenia NOS, psychosis NOS as well as the following ICD-9 diagnoses: unspecified schizophrenia, bipolar affective disorder, manic, were placed in psychosis category. All offenders in this category were proposed to be nonresponsible for their index arson. Subjects with the principal diagnosis of ICD-8 personality disorder (immature, antisocial, narsistic, explosive, NOS) or ICD-9 personality disorder (antisocial, borderline, dependent, mixed type) showed no Axis I diagnosis, except for possible comorbid abuse/dependence disorder, and were placed in the personality disorders category. The alcoholism category included persons with no Axis I or Axis II diagnoses other than substance dependence (ICD-8: episodic excessive drinking, alcoholic addiction, habitual excessive drinking; ICD-9: alcohol dependence syndrome). The organic brain disorder category included persons with severe head injury and personality change as well as those with dementia, while the mood disorders category included patients with nonpsychotic depressive disorders.

### Pure vs. nonpure arsonists

The arson recidivists were then divided into two subgroups based on their previous criminal histories. Pure arsonists were offenders with only arsons in their previous criminal careers, while nonpure arsonists were those who had committed other crimes in addition to arsons (traffic, property and violent offences).

### Pyromania

All 90 forensic psychiatric examination statements were carefully reviewed and pyromania was assessed using the diagnostic criteria of the Diagnostic and Statistical Manual of Mental disorders- Fourth Edition- Text Revision (DSM-IV-TR) [17]. These criteria included tension or affective arousal before the act, fascination with, interest in, curiosity about, or attraction to fire and its situational contexts, as well as pleasure, gratification, or relief when setting fires or when witnessing or participating in their aftermath [17]. Disorders as psychosis, mania, mental retardation, dementia and antisocial personality disorder were used as exclusion criteria according to the DSM-IV-TR.

### Statistics

After description of the sample as a whole, the pure vs. nonpure arsonist subgroups were compared using the nonparametric Mann-Whitney independent samples test for the continuous numeric variables and the chi-square (χ_2_) test for the categorical variables.

## Results

### Characteristics of arson recidivists

The mean age of the arson recidivists was 32.2 (SD 10.9) years. Four out of five of them had never been married. The mean IQ of the subjects was 93.0 (SD 19.7). 16 (17.8%) were mentally retarded (IQ under or 70), in 18 (20%) the principal diagnosis was psychosis, in 47 (52%) personality disorder (immature [n = 11], narsistic [n = 1], explosive [n = 2], antisocial [n = 20], borderline [n = 11], dependent [n = 1], NUD [n = 1]) and in 9 (10%) other diagnoses. A total of 55 subjects (61%) suffered from comorbid alcohol abuse/dependency and 61 persons (68%) committed the index arson under acute alcohol intoxication (mental retardation: 5 /16 persons, psychosis: 11 /18, personality disorder: 38/47, organic brain disorders: 0/2, mood disorders 3/3, alcoholism 4/4 persons).

### Pyromania

After excluding persons with psychosis, mental retardation, organic brain syndrome and antisocial personality disorder (n = 56) 12 of the remaining 34 arson recidivists fulfilled the DSM-IV-TR inclusion criteria for pyromania. However, 9 of these 12 persons were under acute alcohol intoxication during the index arson.

### Pure vs. nonpure arsonists

In all, 43 (48%) of the arson recidivists had only arsons in their previous criminal histories at the time of the evaluation; 15 out of 16 mentally retarded (χ^2 ^= 16.483, df 1, p = 0.000) and 15 out of 18 psychotic persons (χ^2 ^= 11.400, df 1, p = 0.001) belonged to this group of pure arsonists. In contrast, only 12 out of 47 subjects with personality disorder (immature [n = 5], borderline [n = 5], dependent [n = 1], NUD [n = 1]) (χ^2 ^= 19.511, df 1, p = 0.000) and 18 subjects out of 55 (χ^2 ^= 12.840, df1, p = 0.000) with comorbid alcoholism belonged to this group.

The median IQ of the pure arsonists was lower (84.5 [Q1-3 67.25–105.5] vs. 101 [Q1-3 90–110], Z = -3.65, p = 0.000) than of nonpure arsonists. There were no differences in the median ages or marital status between these two subgroups.

## Discussion

In this consecutive sample of arsonists, 22.4% were recidivists, which is similar to the results of some fire-setting studies undertaken among the forensic population [7,11]. The most important diagnostic categories of arson recidivists were personality disorders, psychosis and mental retardation, often with comorbid alcoholism. Nearly 70% of the arsonists were under acute alcohol intoxication during the index crime; these observations were similar to those reported in previous fire-setting studies [7-9].

### The mentally retarded arson recidivists

18% of recidivists were mentally retarded persons and they were almost all pure arsonists. Originally, pathological fire-setting was considered as a phenomenon perpetrated by physically disabled or mentally retarded pubescent girls who had abnormal psychosexual development [18]. Later, due to methodological difficulties, there have been conflicting opinions of whether people with mental retardation are over- or underrepresented in the arsonist population [19]. In a recent prospective follow-up of 61 offenders with intellectual disability, both sex offence and arson were overrepresented offence types and as many as 21.3% of the total population had a history of fire-setting [20]. In contrast to our results, most of the above-mentioned 61 persons with intellectual disability had committed also other offences. A possible explanation for this discrepancy might be that the above mentioned study group included single-setters with an IQ below 80, whereas in our study all the subjects were repeaters and the IQ cutoff point was 70 or below, which generally indicates a significantly impaired intellectual functioning. As far as we know, no previous studies have focused particularly on arson recidivists in this population. Murphy and Clare [21] reported that the three most frequently identified antecedent emotions/events before fire-setting in mentally retarded patients were, in rank order, angry feelings, not being listened to/ attended to, and feeling sad/ depressed. Stereotypical behaviour is frequently found in deeply mentally retarded patients [22], which most of the persons in the present study were not. However, overrepresentation of mentally retarded persons as pure arsonists ma be explained by the idea that they represent persons who express their feelings of anger, frustration and sadness through specific, repetitious action models (in these cases arson), but who do not otherwise express interest in criminal activities.

### The psychotic arson recidivists

20 % of recidivists were psychotic persons and, like mentally disordered patients, they were mostly pure arsonists. A higher than expected prevalence of psychotic disorders among fire-setters was already reported over 60 years ago [23]. Motives of patients with schizophrenia have been reported to be often similar to those observed in non-psychotic persons, with hatred and revenge as the common underlying factors in arson [24]. However, delusions have also been regarded as motives or reasons [25]. In a very large sample of fire-setters as many as 40% of paranoid schizophrenics repeated fire-setting [25]. A large proportion of recidivists set fires to property that is unrelated to them in any way and they also seem to set fires on a very sporadic basis, usually within the context of situational crises [10]. In our sample, most psychotic persons were pure arsonists with no other criminal activities, suggesting, that among psychotic fire-setters there may be different subgroups with various underlying psychological mechanisms leading to fire-setting behaviour. In single-setters the motives may be more instrumental, whereas in recidivists fire-setting is probably a function of flagrant thought disturbance, precipitated by command hallucinations and delusions.

### The arson recidivists with personality disorders

Antisocial personality disorder was the most common personality disorder in the present sample (22 % of recidivists) and, in fact, some of the best predictors of recidivist fire setting are impulsive characteristics [26]. Deliberate fire-setting is one of the diagnostic criteria in conduct disorder, the childhood and adolescent predecessor of adult antisocial personality disorder [17], and self-reported fire- setting is strongly associated with extreme antisocial behaviour in young community adolescents [27]. Recently, adolescent fire-setters were described as aggressive but on the other hand shy and rejected by their peers [28]. All recidivists with antisocial personality disorder were intoxicated during the index crime. In a prospective follow-up study, low central nervous system 5-hydroxyindole acetic acid (CNS 5-HIAA) and 3-methoxy-4-hydrophenylglycol (MHPG) concentrations, typically associated with impulsive antisocial personality and Cloninger type II alcoholism, were associated with both violent recidivist criminal offences and fire-setting [29]. In the present sample all the antisocial fire-setters were nonpure arsonists, and the arson were often aimed at revenge. The underlying motives for these impulsive acts were typically hatred and rage, which increased in severity along with acute substance intoxication. Offenders with severe personality disorder typically begin their criminal careers early and their criminal records are extended. As criminals, their acts appear to be heterogeneous and fire-setting reflects their criminal and unempathic natures [12]. In a study by Repo et al. [8] among arsonists, overall lifetime criminal recidivism was primarily associated with antisocial personality and alcohol dependence.

### The arson recidivists with pyromania

Pyromania is regarded as an impulse-control disorder, but controversy continues over whether the condition should be classified under the impulse-control disorders or, indeed, whether it comprises a separate entity at all. Pyromania was already mentioned in DSM-I, omitted from the DSM-II, but then acknowledged again in the DSM-III [12]. In the DSM-IV-TR a wide range of exclusion criteria such as psychotic states, dementia, mental retardation, monetary gain and political ideologies are presented. The exclusion criteria cover also other criminal acts, rage, revenge and acute intoxication. The phenomenon should also not be observed as pyromania if it is better explained as conduct disorder, antisocial personality disorder or manic state. In our sample, after exclusion of persons with psychosis, mental retardation, organic brain syndrome and antisocial personality disorder, 12 of the arson recidivists fulfilled the inclusion criteria of pyromania. They expressed tension or affective arousal before the act, attraction to and interest in fire, as well as pleasure and release afterwards. However, nearly all these persons were intoxicated during the fire-setting, and they typically mentioned that the tension as well as the affective arousal increased when they were consuming alcohol. Since substance intoxication is one of the exclusion criteria, only three persons with true pyromania were found in the present sample. They all were pure arsonists and, interestingly, all these three men worked as volunteer firefighters and also expressed special interest in fire in other ways as arsonists. In conclusion, using the present strict exclusion criteria for pyromania, the disorder must be regarded as an extremely rare phenomenon as also shown in previous studies [10,11]. The question of substance intoxication as an exclusion criterion should especially be reconsidered.

### Methodological issues

In Finland, an average of about 500–600 arson attempts are made and 100 offenders convicted of arson each year. The proportion of individuals who undergo a forensic psychiatric evaluation compared with all arsonists suspected by the police was estimated to be only 10% [3]. So, the present sample, as well as other previous pretrial samples reported in the literature, is not representative of arsonists in general. The diagnoses were based on several clinical interviews, psychological tests and observation during the two month examination periods. Finnish forensic psychiatric examination statement traditionally includes a paragraph involving a review of the person's previous official criminal history. This information instead of authentic criminal records was used in dividing arsonists into different subpopulations. The strength of the study is that we were able to collect a consecutive sample of male fire-setting recidivists over a period of more than two decades. However, further research is still needed to clarify the important question of fire-setting recidivism.

## Conclusion

The most important diagnostic categories of arson recidivists were personality disorders, psychosis and mental retardation, often with comorbid alcoholism. Psychotic as well as mentally retarded persons with repeated fire-setting behaviour were mostly pure arsonists- persons guilty only of arsons during their criminal careers. Repeating arsonists with personality disorder, in contrast, often exhibited various types of criminal behaviour and arson appeared to be only one expression of a wide range of criminal activity. Using the criteria of the DSM-IV-TR, pyromania is an extremely rare phenomenon. Substance intoxication as an exclusion criterion for pyromania should be reconsidered.

## Competing interests

The author(s) declare that they have no competing interest.

## Authors' contributions

This manuscript was prepared by a multidisciplinary team consisting of:

NL, generated the idea for this study, studied the forensic psychiatric evaluation statements, prepared the manuscript together with the team

MMH, participated in designing the study and in data collection, carried out statistical analyses and prepared the manuscript together with the team

PT, as a neurologists had a substantial contribution in theoretical background concerning mental retardation and participated actively in preparation of this manuscript

MV, supervised and participated with great impact in all stages of preparation of this manuscript

## Pre-publication history

The pre-publication history for this paper can be accessed here:


